# In vivo diffusion-weighted MRI detection of myocardial fibrosis in hypertrophic cardiomyopathy patients

**DOI:** 10.1186/1532-429X-17-S1-P34

**Published:** 2015-02-03

**Authors:** Christopher T Nguyen, Minjie Lu, Xiaoming Bi, Peter Kellman, Debiao Li, Shihua Zhao

**Affiliations:** 1 Biomedical Imaging Research Institute, Cedars-Sinai Medical Center, Los Angeles, CA, USA; 2 Bioengineering, University of California Los Angeles, Los Angeles, CA, USA; 3 State Key Laboratory of Cardiovascular Disease, Fuwai Hospital, Beijing, China; 4 National Center for Cardiovascular Diseases, Chinese Academy of Medical Sciences and Peking Union Medical College, Beijing, China; 5 National Heart, Lung, and Blood Institute, National Institutes of Health, Bethesda, MD, USA; 6 MR R&D, Siemens Healthcare, Los Angeles, CA, USA

## Background

Recent studies have demonstrated the potential of in vivo diffusion-weighted MRI (DWI) in detecting myocardial replacement fibrosis for chronic myocardial infarction [1-3]. Despite the potential of this contrast-free technique, detecting diffuse myocardial fibrosis with DWI has not been established. Current cardiac MRI (CMR) techniques to detect diffuse myocardial fibrosis include late gadolinium enhancement (LGE) [4], pre/post contrast T1 mapping [5], and extracellular volume (ECV) mapping [6]. We propose the application of a recently developed cardiac DWI technique [7] to detect diffuse myocardial fibrosis in HCM patients and compare its performance with established CMR techniques.

## Methods

HCM patients (N = 23) were recruited and consented under Institutional Review Board. All patients were scanned on a 1.5T Siemens Avanto with the following protocol: standard morphological localizers, DWI (3 orthogonal diffusion directions, b = 350 s/mm^2, free breathing) and pre/post contrast T1 mapping (MOLLI, TI_min_ = 100ms, TI_inc_ = 80msec, breath-hold). ADC was calculated using a monoexponential fit. ECV was calculated by fitting pre/post T1 values of the myocardium and blood pool with a collected hematocrit percentage [6]. All images were acquired in the short axis view with matching slice positions. ADC and ECV images were segmented into 6 American Heart Association (AHA) segments. Positive regions for myocardial fibrosis were defined as: ADC > 2.0 μm^2^/ms [1] and ECV > 30% [8]. For ADC and ECV, a two-sample t-test was performed to evaluate the difference between mean values of fibrotic and non-fibrotic regions. To test for agreement in regional detection, Cohen's Kappa test was performed along with calculating sensitivity, specificity, positive predictive value (PPV), and negative predictive value (NPV) using ECV as the gold-standard reference.

## Results

**Figure 1:**
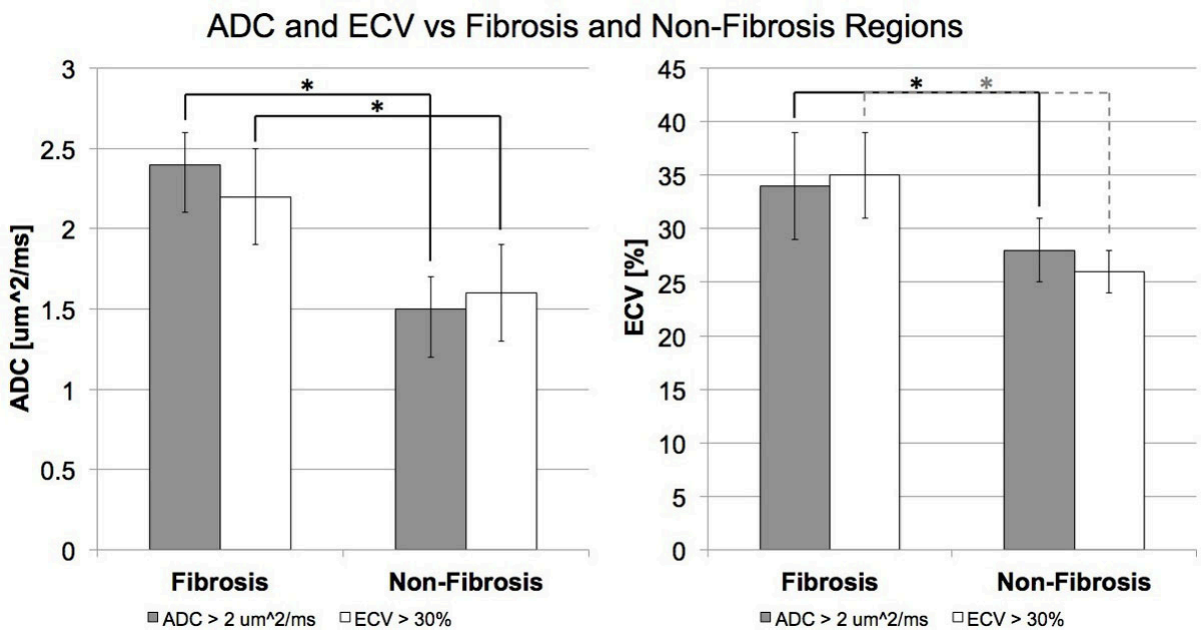
ADC and ECV in fibrosis and non-fibrosis regions defined by either ADC > 2 um^2/ms or ECV > 30% were compared. Both ADC and ECV were significantly (p < 0.01) higher in fibrosis than non-fibrosis regions for both criteria.

**Figure 2:**
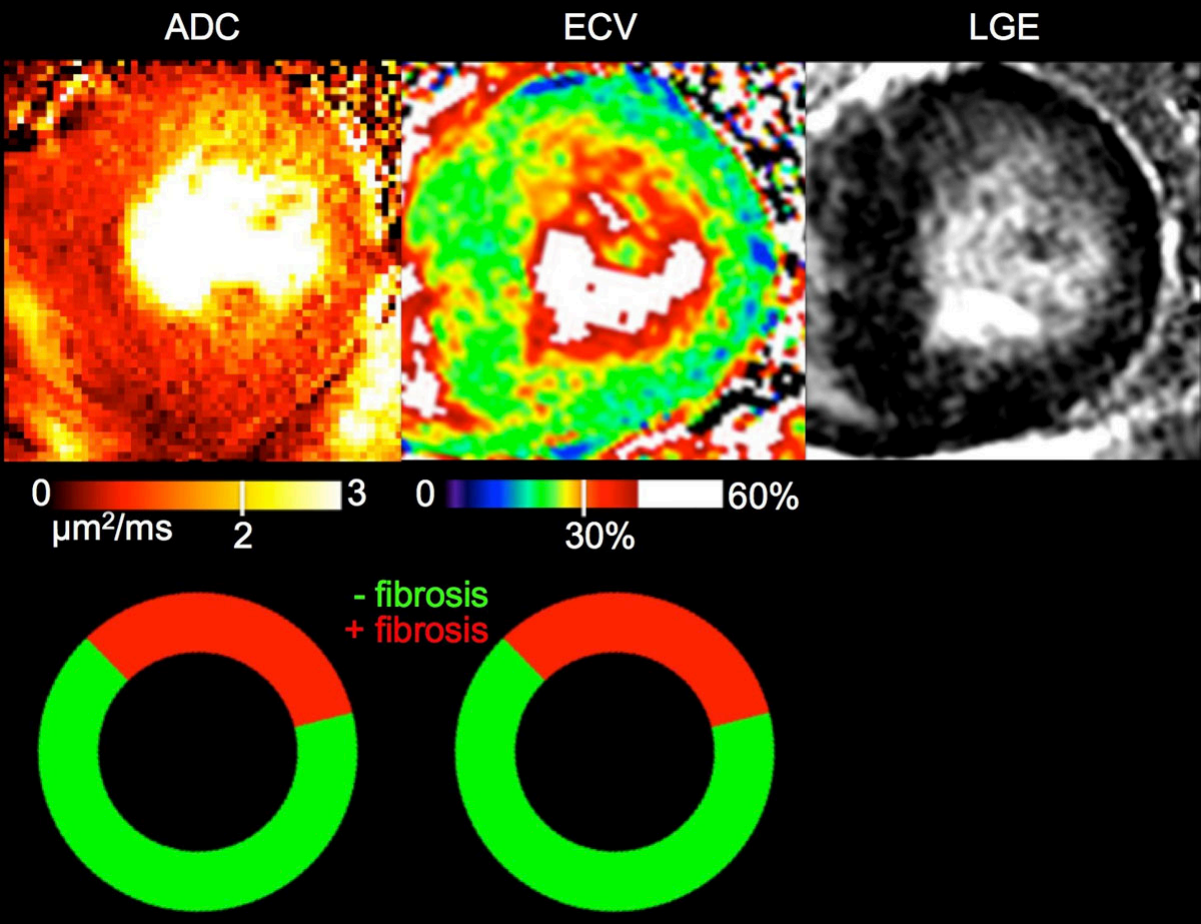
Representative example of ADC and ECV maps with associated AHA wheels. LGE is provided for qualitative reference. Qualitatively, the ADC and ECV maps are in agreement with matching endocardial presentation of fibrosis in the anterior and anteriolateral AHA segments. This is further substantiated quantitatively with excellent agreement in the AHA wheels.

ADC of fibrotic regions (2.4 ± 0.2 μm^2^/ms) was significantly (p<0.01) higher than ADC of non-fibrotic regions (1.5 ± 0.2 μm^2^/ms) (Fig 1, 2). Similarly, ECV of fibrotic regions (35 ± 4%) was significantly (p<0.01) higher than ECV of non-fibrotic regions (26 ± 2%). In fibrotic regions defined by ECV, ADC (2.2 ± 0.3 μm^2^/ms) was again significantly (p< 0.05) higher than ADC of non-fibrotic regions (1.6 ± 0.3 μm^2^/ms). In fibrotic regions defined by ADC criterion, ECV (34 ± 5%) was significantly (p < 0.01) higher than ECV in non-fibrotic regions (28 ± 3%). Regional detection between ADC and ECV of diffuse fibrosis yielded substantial agreement (κ = 0.66) with high sensitivity, specificity, PPV, NPV, and accuracy (0.80, 0.85, 0.81, 0.85, and 0.83, respectively).

## Conclusions

Cardiac DWI is sensitive to diffuse myocardial fibrosis and is capable of characterizing the extent of fibrosis in HCM patients.

## Funding

NIH 1F31EB018152-01A1.
